# Comparing Utilization of Operative versus Awake Laryngoplasty Techniques in the United States Medicare Population: 22‐Year Trends

**DOI:** 10.1002/ohn.70006

**Published:** 2025-08-26

**Authors:** Nour Abdel‐Azim, Emma Thompson, Alexandra Meeter, Mahinaz Mohsen, Roman Povolotskiy, Boris Paskhover, Kenneth Yan, Rachel Kaye

**Affiliations:** ^1^ Department of Otolaryngology Rutgers New Jersey Medical School Newark New Jersey USA; ^2^ Department of Otolaryngology Loma Linda University Health Loma Linda California USA; ^3^ Department of Internal Medicine Temple University Philadelphia Pennsylvania USA; ^4^ Department of Internal Medicine Rutgers New Jersey Medical School Newark New Jersey USA

**Keywords:** current procedural terminology, injection laryngoplasty, laryngoplasty, medicare spending, procedure coding

## Abstract

**Objective:**

Injection laryngoplasty (IL) is performed to correct glottic insufficiency. There has been a purported shift away from operative techniques in favor of awake, in‐office procedures, but no studies comparing utilization include updated current procedural terminology (CPT) coding. We analyzed the usage of operative versus awake injections CPT codes over 2 decades, recognizing that these encompass a broad array of injection procedures.

**Study Design:**

Retrospective database study.

**Setting:**

United States Medicare Population from 2001 to 2022.

**Methods:**

Utilization and reimbursement data compiled by the US Centers for Medicare & Medicaid Services were queried for CPT codes encompassing awake injections (31513, 31573, 31574), operative ILs (31570, 31571), and operative medialization laryngoplasties (31588, 31591).

**Results:**

10,186 injections were performed in 2022, a 195% increase from 2001. Awake injections grew by 412.77%, while operative injections grew by 134.75%. Operative injections are still more common (79.4% in 2001; 64.24% in 2022) and population‐adjusted use of 31571 grew between 2001 and 2021, while 31570 decreased. Since its adoption in 2017, awake IL code 31574 increased by 66.6%. From 2001 to 2006, the annual growth rate of awake injections was significantly lower than that of operative IL (*P* < .0001). From 2017 onwards, the awake IL growth rate was significantly higher than operative injections (*P* = .020).

**Conclusions:**

Awake injection utilization increased over the 22‐year period, as introduction of code 31574 coincided with a relative decline in operative and an increase in awake IL. Otolaryngologists may be implementing awake injections due to reduced recovery time and introduction of more specific CPT codes.

Injection laryngoplasty (IL) is a well‐established procedure used to correct glottal insufficiency, which can result from fibrosis, paresis, atrophy, or vocal fold immobility.^1^, ^2^ By achieving vocal fold medialization, IL improves dysphonia, dysphagia, and patients' quality of life.^3^, ^4^ While IL has been performed for over a century, current literature asserts that the setting has shifted over time, with an increase in procedures that are performed awake and in‐office rather than under general anesthesia in the operating room.^2^, ^5^, ^6^ This is especially relevant in light of the broader shift to minimally invasive procedures and efforts to increase operating room efficiency.

“Operative” and “awake” ILs are defined by their Concurrent Procedural Terminology (CPT) codes, which are utilized to charge for medical procedures. Data on utilization of these codes is publicly available for patients enrolled in Medicare. Although these codes are essential tools for tracking procedural trends, they have historically lacked specificity. CPT codes 31570 and 31571 are used for ILs as performed in an operating room under sedation or general anesthesia utilizing a rigid laryngoscope or microscope for guidance, but these codes also encompass injections beyond vocal fold augmentation.^1^, ^7^ These broader indications include corticosteroid injections and botulinum toxin injections, which address a wider range of laryngeal pathologies. Specifically, 31570 describes procedures utilizing a direct view with a laryngoscope for the injection, while 31571 covers injections performed using direct laryngoscopy with the assistance of a telescope or an operative microscope.^8^ Thus, while CPT codes can be used to obtain valuable and standardized data, their lack of exactitude presents challenges in studying IL trends.

Similarly, “awake injections,” covered by CPT codes 31513 and 31574, include many injections performed through indirect laryngoscopy (peroral, flexible nasofiberoptic) and percutaneous injections. The injections can be administered transorally, percutaneously, or directly via a working channel flexible laryngoscope. Typically, awake injections occur in outpatient or office‐based settings and utilize local anesthesia. However, awake injections may also occur in operating rooms, with or without sedation, depending on patient‐specific risks or procedural complexity. Despite the theorized rapid adoption of awake injections, 31513, a code implying use of a laryngeal mirror rather than a laryngoscope, was the only code available for awake injections until 2017.^2^ This nonspecific code limited precise tracking of awake ILs.

One of the proposed justifications for the shift towards awake injections is the reduced need to undergo general anesthesia and associated cost‐savings, with studies suggesting that awake IL is more cost‐effective for patients and providers.^9^ Savings are attributed to reduced staffing requirements and lower costs of anesthesia, and facility fees.^10^, ^11^ Additionally, awake IL may expedite time to treatment due to increased flexibility in scheduling nonoperative procedures. Rapid access to IL offers benefits—1 metanalysis found that performing IL within 6 months of experiencing unilateral vocal fold weakness reduces the need for a future thyroplasty, sparing additional expenses and complications.^12^ The safety and efficacy of awake ILs has been established, with minimal complications, pain, and comparable outcomes to operative ILs.^13^, ^14^, ^15^, ^16^, ^17^, ^18^

Type I thyroplasty, or “medialization laryngoplasty,” medializes the vocal folds. Thyroplasty augments the vocal folds to optimize their medial positioning and thereby acts as the permanent treatment for most pathologies that IL addresses.^19^ There are 2 associated CPT codes for thyroplasty, 31588 and 31591. 31591 was introduced in 2017 and effectively replaced 31588, which was a “thyroplasty not otherwise specified” code and is an umbrella term encompassing several forms of thyroplasty.^20^

An important shift occurred in 2017 when the American Medical Association added the 31574 CPT code for percutaneous or transoral unilateral injections guided by laryngoscopy, offering an appropriate code for awake ILs.^20^ Prior to this, only code 31513 was available, limiting its use and the ability to analyze its implementation. Accordingly, no studies have examined the impact of the introduction of code 31574 on awake IL utilization. The most recent study only assessed CPT codes through 2012, not accounting for the 2017 update in CPT codes for IL.^21^, ^22^

This study's primary objective is to investigate the trends in usage of CPT codes that include ILs within the US Medicare population from 2001 to 2016 and from 2017 to 2022, with a focus on the trends of awake, in‐office procedures and the impact of updated CPT codes. The secondary objective is to analyze trends in nonoperative and operative injections, using the utilization of operative medialization laryngoplasty (ML) as a benchmark to account for potential changes in the incidence or prevalence of glottic insufficiency.

## Methods

Data were collected from the Centers for Medicare & Medicaid Services (CMS) website under the section “Research, Statistics, Data & Systems.” The Part B National Summary datasets were queried using current CPT codes to extract the total number of annual procedures performed for the years 2001 to 2022. The total annual reimbursement amount (in US dollars), as listed in the Part B National Summary dataset, include professional fees for both surgeons and physician assistants, as well as coinsurance and deductible amounts, representing the total net payment for all services performed each year.

CPT codes 31513 and 31574 were categorized as awake, in‐office injections, while codes 31570 and 31571 were categorized as operative injections performed under general anesthesia (Table 1). 31570 and 31571 are not specific to ILs and include injections that are often performed concurrently with other laryngeal procedures.^8^

**Table 1:** CPT Codes by Description

Procedure	CPT code	Code description
Awake injections	31513	Laryngoscopy, indirect; with vocal cord injection
	31574	Laryngoscopy, flexible; with injection(s) for augmentation
Awake flexible injections	31573	Laryngoscopy, flexible; with therapeutic injection(s), any site
	31574	Laryngoscopy, flexible; with injection(s) for augmentation
Operative injections	31570	Laryngoscopy, direct, with injection into vocal cord(s), therapeutic
	31571	Laryngoscopy, direct, with injection into vocal cord(s), therapeutic; with operating microscope or telescope
Medialization laryngoplasty	31588	Laryngoplasty not otherwise specified
	31591	Laryngoplasty, medialization, unilateral

Similarly, the awake IL code 31513, used prior to 2017, was not specific to flexible endoscopic injection augmentations. Codes 31588 and 31591 were categorized as ML, the permanent treatment for glottic insufficiency. ML was used as a benchmark for comparative growth and to ensure that observations were not due to changes in prevalence of glottic insufficiency within the general population. The last available data for code 31588 was in 2016 as this code was deleted and subsequent MLs (2017 and after) were reported under CPT code 31591.

Two time periods, 2001 to 2016 and 2017 to 2022, were examined to evaluate the effects of the new CPT codes released in 2017 (31574, 31591) on coding and procedural practices. Total annual procedure counts were standardized to the number of US Medicare enrollees for each year, producing the time‐normalized “procedures per enrollee.” Historical enrollment figures were obtained from the Medicare Enrollment Report file. All dollar values were adjusted for inflation relative to the 2021 average consumer price index.

Provider characteristics and state‐level data were collected from the “Medicare Physician & Other Practitioners—by Provider and Service” section of the CMS website for the years 2013 to 2022. Data for CPT codes 31513, 31570, 31571, 31574, 31588, and 31591 were extracted. “Procedures per 1000 enrollees” and “Procedures per enrollee” were calculated utilizing the number of US Medicare enrollees for each year. “Payment per procedure” was calculated utilizing the number of procedures and the average Medicare payment amount from the data set. Data were filtered by state to create heat maps.

Data extraction, processing, and visualization were performed with Microsoft Excel. To compare growth rates, linear regression analysis with slope comparison was performed using GraphPad Prism. All statistical tests were conducted with an *α* level of 0.05. A nonhuman research determination was approved by the institutional review board at Rutgers University, New Brunswick, New Jersey (Pro2022000491).

## Results

### National Level

**Figure 1:**
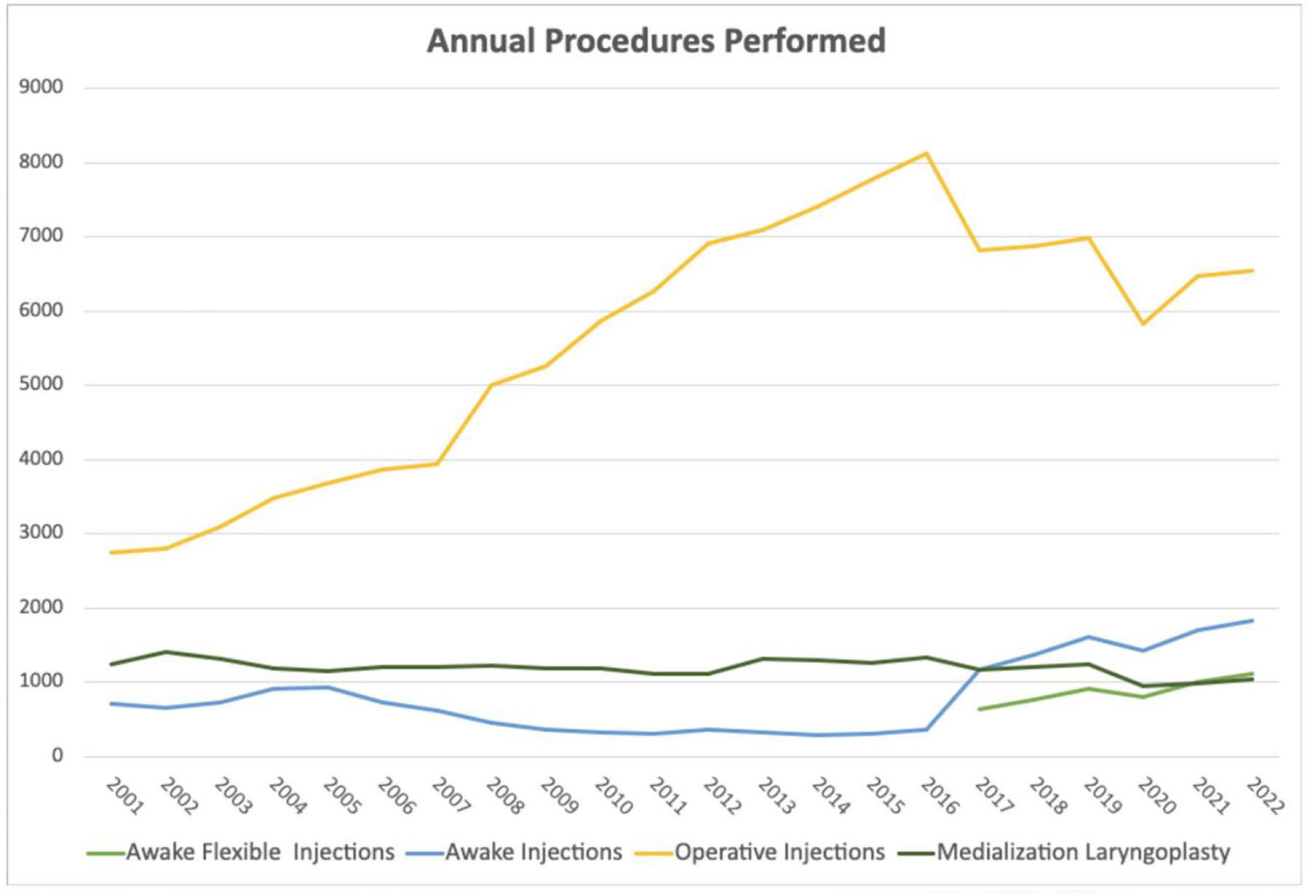
Number of Medicare claims submitted annually for procedure type, from 2001 to 2022. Line graph depicting the yearly number of Medicare claims for awake injections, awake flexible injections, operative injections, and ML, from 2001 to 2022.

**Figure 2:**
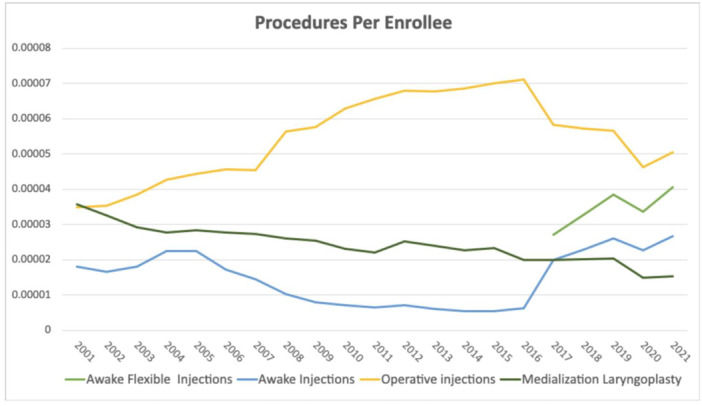
Annual number of procedures per Medicare enrollee from 2001 to 2021. Line graph depicting the yearly number of procedures per Medicare enrollee from 2001 to 2022 for awake injections, awake flexible injections, operative injections, and ML.

**Figure 3:**
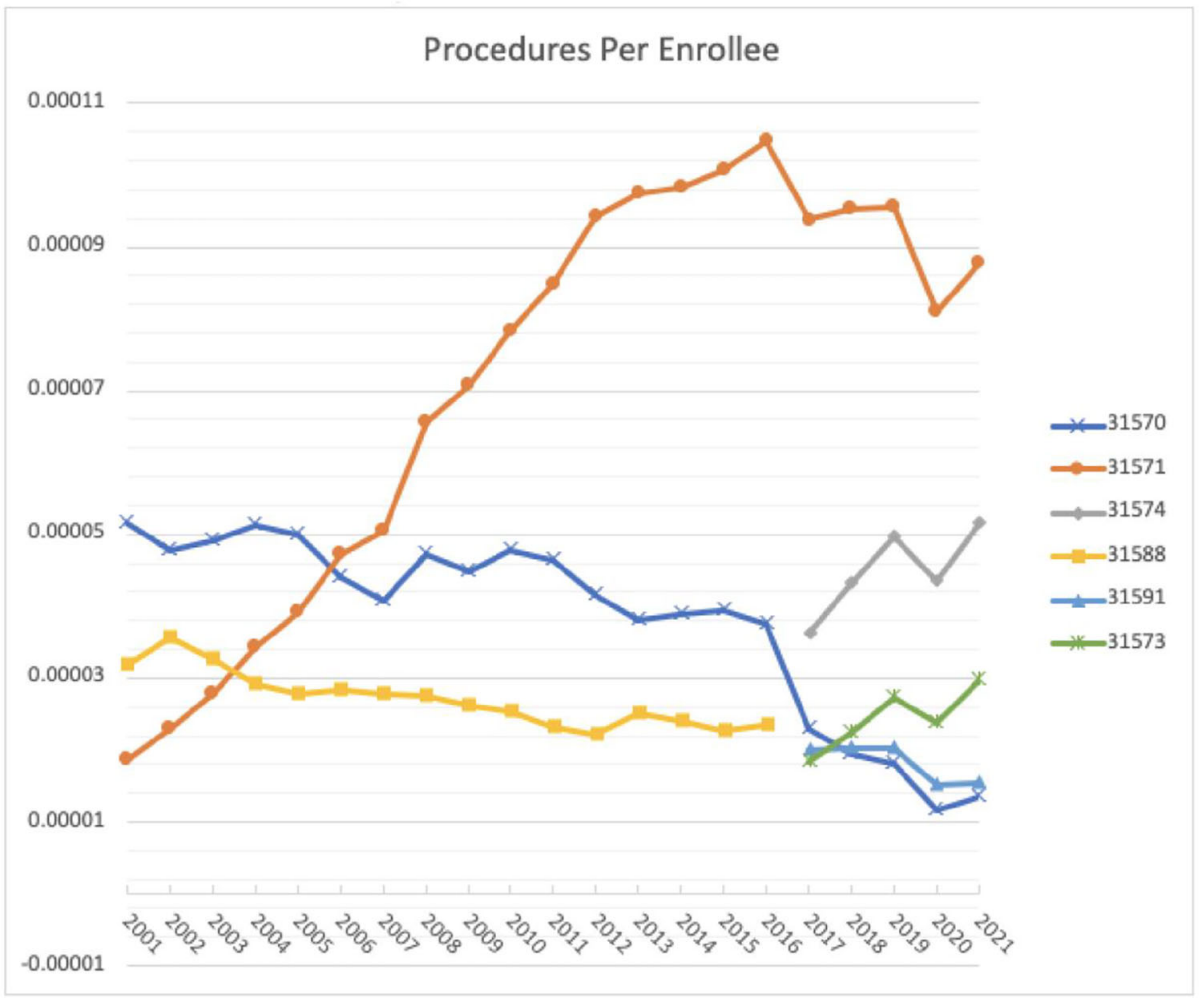
Procedures per enrollee. Line graph depicting the yearly number of procedures by current procedural terminology codes for awake injections, awake flexible injections, operative injections, and ML, from 2001 to 2022.

From 2001 to 2016, utilization of awake injections declined significantly by 49.93% (65.6% decrease per enrollee, *P* = .0004), while utilization of operative injections increased by 196.17% over the same time period (103.48% increase per enrollee, *P* < .0001, Figures 1 and 2). The difference in the rate of change of utilization between awake and operative injection codes was also significantly different from 2001 to 2016 (*P* < .0001). Operative injections were the most common laryngoplasty procedure performed between 2001 and 2016, representing 90.8% of injections. Regarding the rates of procedures per Medicare enrollee, 31571 increased significantly from 2001 to 2016, while the other operative code, 31570, and the awake injection code, 31513, both declined significantly (*P* < .0001, Figure 3).

This finding inverts from 2017 to 2022, after introduction of the new awake IL code 31574. During that time, the increase in use of awake IL and injection codes was significantly higher than operative injection codes (*P* = .016). Utilization of awake injection codes grew significantly by 35.91% (33.36% increase per enrollee from 2017 to 2021, *P* = .013) whereas operative injections use fell by 4.06% (*P* = .34). The utilization of the newly introduced code 31574 increased significantly by 66.6% from 2017 to 2022, representing a 42.4% increase per enrollee from 2017 to 2021 (*P* = .009). Over the same time period, utilization of operative injections code 31570 decreased significantly by 34.68% (40.92% decrease per enrollee from 2017 to 2022, *P* = .03) and use of 31571 did not significantly change (*P* = .95). From 2016 to 2017, the year of introduction of the 31574 code, utilization of awake injection codes increased by 556.18% (539.61% per enrollee). When comparing combined awake flexible injections (31573, 31574) and operative codes (31570, 31571) from 2017 to 2022, awake injections grew by 76.9% while operative injection codes declined by 4.1% (*P* = .0028).

Over the total period, regression analysis showed use of awake injections grew significantly by 412.7% from 2001 to 2022, with a 193.4% increase per enrollee from 2001 to 2021 (*P* = .0003). Operative injections also significantly increased by 138.72% (*P* < .0001), at 44.64% per enrollee. However, the absolute number of operative injections was consistently higher every year compared to awake injections, comprising over 79.4% of all injections in 2001 and 64.2% in 2022.

A total of 26,376 operative MLs were performed from 2001 to 2022. On regression, the number of MLs performed each year decreased significantly from 2001 to 2022 (*P* = .02) (52.0% decrease per enrollee from 2001 to 2021). However, regressions for subperiods of 2001 to 2016 and 2017 to 2022 did not find significant changes in utilization over either shorter interval (*P* = .8761 and *P* = .1375).

Average annual Medicare reimbursement data were used as a surrogate measure for procedural costs (Table 2). Code 31513 for awake injections initially cost $175.19 per procedure in 2001, subsequently stabilizing in 2007 until its most recent average price point at $86.28 (54.1% decrease in average cost per procedure). This represents a significant decrease in reimbursement (*P* < .001). Code 31574 cost $537.44 on average in 2017, increasing significantly by 17.52% until 2021 when it cost $631.58 (*P* = .031). The operative codes 31570 and 31571 were initially priced at just above $300 in 2001. Both fell significantly, 31570 by 17.9%, while 31571 fell by 24.76% (*P* < .0001 and *P* = .0001).

**Table 2:** Trends in Payment Per Procedure Adjusted to 2021 Inflation Measure

	2001	2016	2017	2021	Change 2001‐2016	Change 2017‐2021
Operative injections						
31570	$328.40	$295.88	$275.73	$269.60	−9.90%, *P* = .004*	−2.23%, *P* = .67
31571	$323.20	$263.10	$261.98	$243.16	−18.60%, *P* = .03*	−7.18%, *P* = .018*
Awake injections						
31513	$175.19	$104.25	$98.6	$86.28	−40.50%, *P* < .0001*	−12.50%, *P* = .055
31574	N/A	N/A	$537.44	$631.59	N/A	+17.52%, *P* = .03*

* Statistically significant *P*‐value.

### State Level Code Utilization and Payments

From 2017 to 2021, the average payment per procedure among states for an awake injection was $17.66, significantly higher than for an operative injection at $6.05 (*P* < .001). Average procedures per enrollee were also significantly different, at $0.733 per million enrollees compared to $1.61 per million enrollees for operative injections (*P* < .001). Since 31574 was not introduced until 2017, averages were calculated for 2013 to 2016, 2017 to 2021, and 2013 to 2021 for only the operative injections code 31571. The average payment per procedure among states for operative injections was not significantly lower from 2013 to 2016 compared to 2017 to 2021 ($5.81 vs $6.09, *P* = .15). Notably, the average procedure per enrollee of operative injection across all states significantly declined after the introduction of the new 31574 code for awake IL between 2013 to 2016 to and 2017 to 2021 (1.84E−06 vs 1.61E−06, *P* < .001).

**Figure 4:**
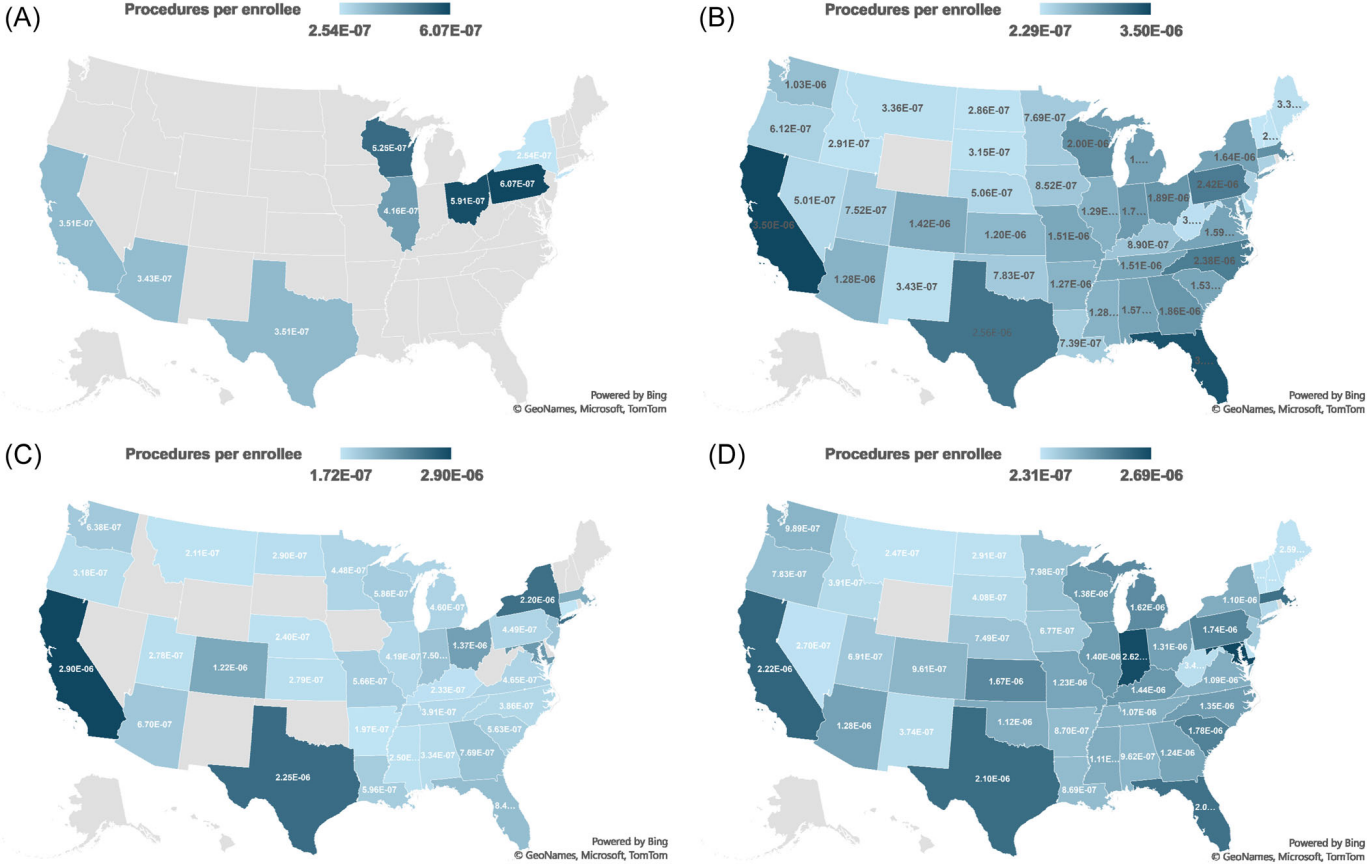
Procedures per Enrollee by State. Heat maps showing procedure variations per enrollee. Darker colors indicate more procedures, lighter colors indicate fewer. (A) Awake Injections from 2013–2016. (B) Operative Injections from 2013–2016. (C) Awake Injections from 2017–2021. (D) Operative Injections from 2017–2021.

**Figure 5:**
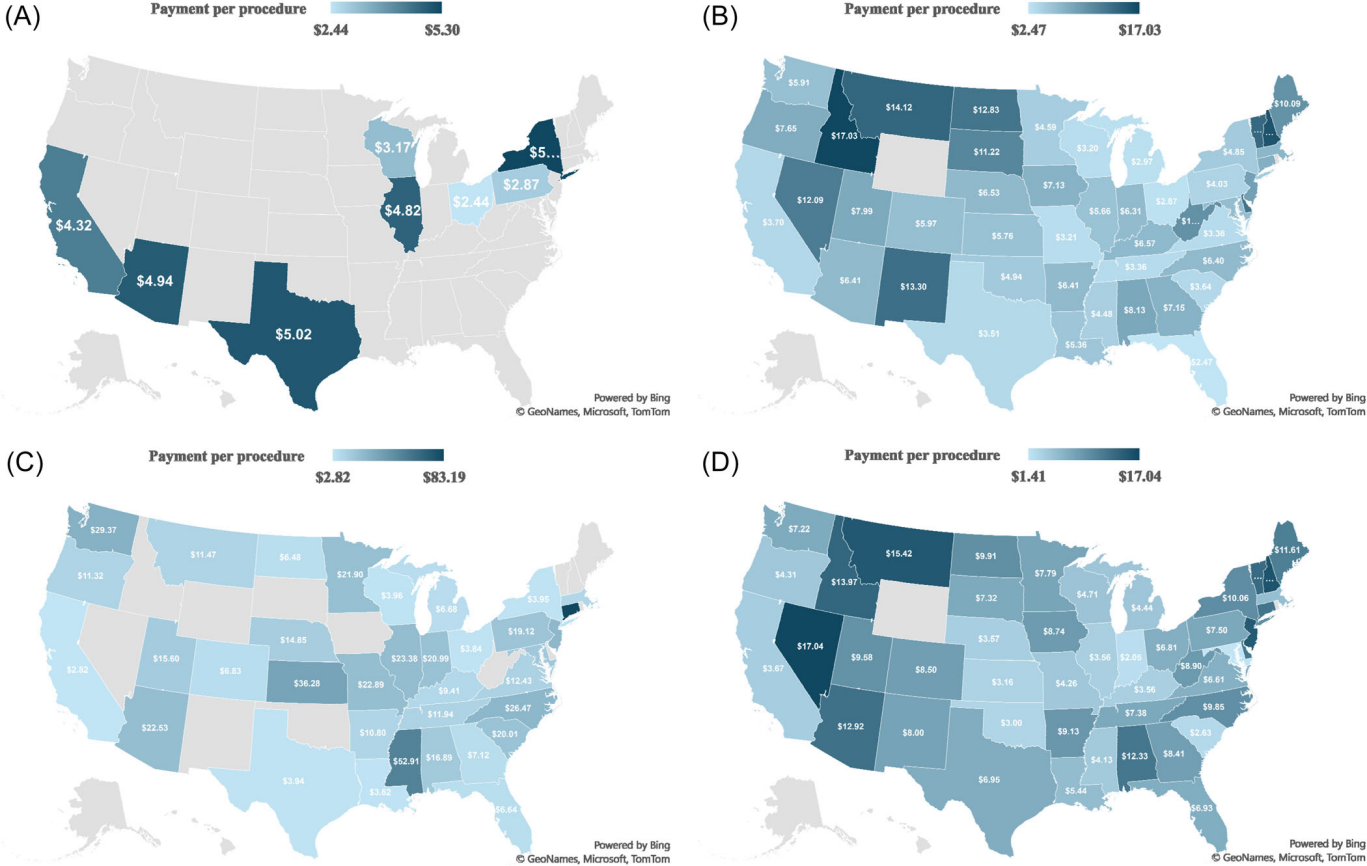
Payment per procedure by state. Heat maps show geographic variations in payment. Darker colors indicate higher payments; lighter colors indicate lower payments. (A) Awake Injections from 2013–2016. (B) Operative Injections from 2013–2016. (C) Awake Injections from 2017–2025. (D) Operative Injectionsfrom 2017–2021.

When analyzing state‐based utilization, operative injections utilized per enrollee from 2013 to 2016 was highest in California, Texas, and Florida, while awake injections were most utilized in Pennsylvania, Ohio, and Wisconsin (Figure 4). Over this same period, payment per procedure for operative injections was highest in Idaho, New Hampshire, and Montana, while awake injections had the highest payment per procedure in New York, Texas, and Arizona (Figure 5). From 2017 to 2021, utilization of operative injection per enrollee was highest in Maryland, Indiana, and California, while awake injection utilization was highest in California, Texas, and New York. Operative injections had the highest payments in Nevada, New Hampshire, and Montana, while awake injections had the highest payments in Connecticut, Mississippi, and Nebraska.

### Provider Characteristics

Provider characteristics were also queried for codes 31574 and 31571. Most providers for both codes were MDs (92.8% and 88%) and male (69% and 72.7%, Tables 3 and 4). Both procedures were overwhelmingly performed by otolaryngologists (99.5% and 93%, respectively) and by individuals rather than organizations (96.8% and 94.7%, respectively). 90.2% of operative injections and 42% of awake injections were performed in facilities; the rest were performed in non‐facility settings.

**Table 3:** Provider Characteristics for Awake Injections (2013‐2022)

	N (%)	Procedures per 1000 enrollees (±SD)	Payment per procedure (±SD)	Payment per 1000 enrollees (±SD)	Allowed amount per procedure (±SD)	Charge amount per procedure (±SD)
Credentials						
MD	425 (95.7%)	3.6E−7 (±2.2E−8)	$575.38 (±458)	$0.00872 (±0.00741)	$725.21 (±573)	$3097.40 (±2334)
MD, MBA	7 (1.5%)	2.6E−7 (±5.2E−8)	$371.70 (±288)	$0.00648 (±0.00506)	$606.86 (±515)	$917.93 (±91)
MD, MS	1 (0.2%)	N/A^a^	$1171.66 (±0)	N/A^a^	$1466.29 (±0)	$6921.43 (±0)
MD, PhD	4 (0.9%)	2.7E−7 (±3.1E−8)	$811.57 (±411)	$0.01151 (±0.00698)	$1012.47 (±512)	$7071.06 (±2859)
Unspecified	7 (1.6%)	2.6E−7 (±5.7E−8)	$480.76 (±411)	$0.01037 (±0.00681)	$606.86 (±515)	$3767.67 (±3045)
Sex	N (%)	Procedures per 1000 enrollees (±SD)	Payment per procedure (±SD)	Payment per 1000 enrollees (±SD)	Allowed amount per procedure (±SD)	Charge amount per procedure (±SD)
Female	134 (30%)	3.3E−7 (±1.6E−8)	$574.54 (±456)	$0.00888 (±0.00729)	$722.91 (±569)	$3190.08 (±1981)
Male	310 (70%)	3.7E−7 (±2.4E−8)	$573.98 (±459)	$0.00867 (±0.00742)	$723.89 (±573)	$3086.87 (±2540)
Provider entity	N (%)	Procedures per 1000 enrollees (±SD)	Payment per procedure (±SD)	Payment per 1000 enrollees (±SD)	Allowed amount per procedure (±SD)	Charge amount per procedure (±SD)
Individual	444 (96.9%)	3.6E−7 (±2.2E−8)	$574.15 (±457)	$0.00874 (±0.00738)	$723.59 (±571)	$3118.02 (±2386)
Organization	14 (3.1%)	6.6E−7 (±2.4E−8)	$387.36 (±55)	$0.00630 (±0.00109)	$486.31 (±68)	$5268.11 (±2633)
Provider specialty	N (%)	Procedures per 1000 enrollees (±SD)	Payment per procedure (±SD)	Payment per 1000 enrollees (±SD)	Allowed amount per procedure (±SD)	Charge amount per procedure (±SD)
Otolaryngology	442 (99.5%)	3.6E−7 (±2.2E−8)	$576.25 (±457)	$0.00878 (±0.00738)	$726.20 (±571)	$3117.61 (±2391)
Pediatrics	2 (0.5%)	2.1E−7 (±2.2E−8)	$387.36 (±55)	$0.00172 (±0.00003)	$486.31 (±68)	$5268.11 (±2633)
Procedure setting	N (%)	Procedures per 1000 enrollees (±SD)	Payment per procedure (±SD)	Payment per 1000 enrollees (±SD)	Allowed amount per procedure (±SD)	Charge amount per procedure (±SD)
Facility	191 (41.7%)	3.5E−7 (±1.8E−8)	$155.90 (±70)	$0.00256 (±0.00117)	$200.49 (±86)	$2208.64 (±1884)
Non‐facility	267 (58.3%)	3.8E−7 (±2.6E−8)	$863.55 (±370)	$0.01374 (±0.00622)	$1085.36 (±463)	$3881.28 (±2521)

^a^ No procedures were performed in this subset from 2013 to 2021.

**Table 4:** Provider Characteristics for Operative Injections (2013‐2022)

Credentials	N (%)	Procedures per 1000 enrollees (±SD)	Payment per procedure (±SD)	Payment per 1000 enrollees (±SD)	Allowed amount per procedure (±SD)	Charge amount per procedure (±SD)
DO	19 (2.1%)	3.7E−7 (±1.6E−8)	$150.57 (±45)	$0.002572 (±0.0008)	$192.12 (±60)	$586.61 (±331)
MD	845 (93.5%)	4.3E−7 (±2.7E−8)	$158.46 (±50)	$0.002781 (±0.0009)	$201.90 (±64)	$1160.76 (±912)
MD, MBA	3 (0.33%)	2.3E−7 (±2.2E−8)	$106.73 (±26)	$0.001968 (±0.0000)	$139.10 (±37)	$1088.00 (±0)
MD, MHS	1 (0.11%)	N/A*	$149.92 (±20)	N/A^a^	$187.83 (±25)	$950.00 (±0)
MD, MS	1 (0.11%)	2.0E−7 (±9.4E−8)	$149.92 (±20)	$0.002589 (±0.0006)	$187.83 (±25)	$1793.50 (±753)
MD, PhD	29 (3.2%)	4.8E−7 (±3.3E−8)	$164.87 (±33)	$0.002925 (±0.0007)	$209.32 (±41)	$1321.07 (±753)
Unspecified	4 (0.44%)	5.0E−7 (±2.5E−8)	$249.64 (±76)	$0.004430 (±0.0015)	$319.26 (±99)	$1884.40 (±36)
Sex						
Female	205 (22.7%)	3.8E−7 (±1.9E−8)	$149.88 (±40)	$0.002619 (±0.0007)	$191.17 (±51)	$1170.43 (±578)
Male	698 (77.3%)	4.4E−7 (±2.8E−8)	$161.29 (±52)	$0.002835 (±0.0010)	$205.45 (±67)	$1154.30 (±969)
Provider entity						
Individual	903 (95.1%)	4.2E−7 (±2.7E−8)	$158.70 (±50)	$0.002786 (±0.0009)	$202.20 (±64)	$1157.96 (±896)
Organization	47 (4.9%)	3.0E−7 (±1.0E−8)	$835.31 (±113)	$0.014264 (±0.0016)	$1050.27 (±140)	$5703.40 (±4746)
Provider specialty						
Otolaryngology	880 (97.5%)	4.2E−7 (±2.6E−8)	$157.31 (±48)	$0.002765 (±0.0009)	$200.53 (±62)	$1171.75 (±903)
Pediatric medicine	8 (0.89%)	3.1E−7 (±6.9E−8)	$121.75 (±19)	$0.002757 (±0.0001)	$154.08 (±21)	$618.23 (±47)
Emergency medicine	5 (0.55%)	2.4E−7 (±3.6E−8)	$168.31 (±10)	$0.002765 (±0.0009)	$212.31 (±13)	$610.00 (±0)
Neurology	10 (1.1%)	9.8E−7 (±2.0E−8)	$306.04 (±6)	$0.002073 (±0.0003)	$383.25 (±7)	$650.00 (±0)
Procedure setting						
Facility	857 (90.2%)	4.0E−7 (±2.3E−8)	$184.94 (±163)	$0.003268 (±0.0029)	$234.60 (±204)	$1429.19 (±1764)
Non‐facility	93 (9.8%)	3.5E−7 (±1.5E−8)	$258.89 (±43)	$0.003427 (±0.0005)	$332.24 (±52)	$955.73 (±457)

_a_ No procedures were performed in this subset from 2013 to 2021.

## Discussion

This study evaluated trends in utilization of codes that include operative and awake injections in the US Medicare population from 2001 to 2016 and 2017 to 2022, with focus on the impact of introducing code 31574 for awake IL in 2017. From 2001 to 2016, utilization of operative injection codes (31570 and 31571) dominated, while awake injections (code 31513) declined in use despite established cost efficacy.^11^ This is also reflected in state‐level analyses.

This likely indicates lack of CPT codes specific to ILs. Without proper coding, physicians cannot bill accurately, contributing to underpayment or denial of claims. Ambiguity in billing and challenging reimbursement likely discouraged broader adoption of awake IL, as highlighted by the American Academy of Otolaryngology–Head and Neck Surgery (AAO–HNS) in 2014 when they recommended utilizing code 31599 for awake ILs due to lack of alternatives.^8^, ^14^ CPT code 31599 for “unlisted laryngeal procedure” encompasses numerous procedures and was excluded from analysis.

The introduction of code 31574 provided an accurate billing mechanism for awake injections with flexible laryngoscopy.^15^ Its rapid uptake from 2016 to 2017 likely reflected improved reimbursement for preferred procedural technique. The significant growth of 31574 from 2017 to 2022, combined with the decline of operative codes (31570, 31571), supports the hypothesis by Rosen et al that awake IL was initially poorly adopted due to lack of appropriate coding.^22^ Our finding that flexible injection codes (31573, 31574) grew significantly between 2017 and 2022 bolsters this and the inclusion of 31573 helps capture the shift towards awake/flexible procedures in general.

Still, operative injections comprise the majority of procedures performed annually. This likely reflects the broad range of indications, therapeutic use (botulinum toxin, steroid, etc), and use in conjunction with other concomitant procedures. Unfortunately, CMS data lacks encounter level‐detail to exclude such cases. As 31570 and 31571 include therapeutic and augmentation injections, we included 31573 to offer a similar comparison. While this may introduce heterogeneity due to the range of injectate, examining the combined trends offers insight into practice patterns. The comparison was significant with total awake/flexible injection utilization (31573, 31574) increased from 2017 to 2022, while the combined 31570 and 31571 declined, reinforcing a broader shift towards awake techniques.

Ambulatory surgery center closures during the COVID‐19 pandemic likely accelerated the shift towards awake injections.^11^ In 2020, utilization of CPT code 31570 dropped by 33.2% (34.7% per enrollee), possibly due to pandemic‐related restrictions. Awake procedures require less equipment and staff, becoming particularly useful during times of limited operating room access. The trend toward increased awake IL performance was already established prior to 2020, emphasizing that these changes are part of a broader shift in procedural settings due to updated CPT coding, although, procedural disruptions caused by COVID‐19 likely amplified the preference for office‐based injections.

Significant state‐level variations in awake and operative injection utilization may reflect regional differences in provider practice patterns, local reimbursement dynamics, patient demographics, or healthcare access. These geographic disparities could be better understood with further investigation to identify and address barriers to broader adoption of awake injections.

Thyroplasty did not significantly decrease between 2001 and 2016 or 2017 and 2022. This is likely due to either a cumulative effect from changing practices over time or due to less data variability within each shorter subperiod. The lack of subperiod change indicates that our other findings were not due to an underlying shift in pathology severity or prevalence.

Though the rate of growth of operative injections was significantly lower than that of awake injections between 2001 and 2022, operative procedures still comprised the majority of injections in recent years. Operative injections were the majority across all time periods, despite trends showing decreasing utilization. This may be largely due to both operative IL codes (31570 and 31571) covering other injections via direct laryngoscopy under anesthesia, including injection of steroids or botox.^8^ We suspect that the reported frequencies of 31570 and 31571 are significantly inflated relative to actual operative injections performed. These findings also highlight a need for the adoption of more specific CPT codes that distinguish operative ILs from other operative laryngeal injections.

Differences in average Medicare payment per procedure may also influence utilization. While inflation adjusted reimbursement for 31513, 31570, and 31571 significantly declined from 2001 to 2016, only 31574 saw a significant increase in payment per procedure performed throughout 2017 to 2021. Reimbursement for 31571 significantly decreased from 2017 to 2021, while 31570 and 31513 did not significantly decrease. Fair compensation likely improves cost‐efficacy, enabling providers to perform procedures. This may partially explain the increased utilization of awake injections. Reduced facility fees for awake injections, and reduced anesthesia/support staff fees, allows for cost‐savings. Given the challenges posed by CPT code limitations, if updates in procedural coding explicitly distinguish ILs from non‐augmentative injections, this would enhance the ability to assess utilization trends.

## Limitations

There are several limitations to this study. Our data represents utilization of procedures in patients enrolled in US Medicare Part B who are predominantly ≥65 years of age. Our findings may not generalize to younger populations or patients outside of the Medicare system. The lack of specificity inherent in the CPT codes for both awake (31513, pre‐2017), and operative injections (31570, 31571) also complicates precise interpretation. This may have resulted in an overestimation of augmentation‐specific procedures and introduced difficulty in accurately assessing procedural trends.

The CPT codes we included might not encompass all injections performed. Prior to code 31574, AAO–HNS recommended using 31599 for unlisted procedures such as percutaneous injections using flexible endoscopic guidance, resulting in underrepresentation of these procedures in our analysis.^23^ Despite these limitations, our study provides insights into general trends and highlights important areas for improvement in procedural coding specificity.

## Conclusions

Utilization of awake, nonoperative vocal fold injections has increased in the US Medicare population since the introduction of CPT code 31574. However, operative injections comprise the majority of procedures performed. Our results suggest providers are preferentially shifting towards awake injections, possibly due to lower costs, patient outcomes, and the introduction of more descriptive and adequate CPT codes.

## Author Contributions

**Nour Abdel‐Azim**, MD: design, analysis, writing; **Emma Thompson**, BA: design, analysis, writing; **Alexandra Meeter**, MD: writing; **Mahinaz Mosen**, MD: writing; **Roman Povolitsky**, MD: writing, reviewing; **Boris Paskhover**, MD: design, reviewing; **Kenneth Yan**, MD, PhD: design, writing, reviewing; **Rachel Kaye**, MD, FACS: design, writing, reviewing.

## Disclosures

### Competing interests

None.

### Funding source

None.
